# Explaining the Host-Finding Behavior of Blood-Sucking Insects: Computerized Simulation of the Effects of Habitat Geometry on Tsetse Fly Movement

**DOI:** 10.1371/journal.pntd.0002901

**Published:** 2014-06-12

**Authors:** Glyn A. Vale, John W. Hargrove, Philippe Solano, Fabrice Courtin, Jean-Baptiste Rayaisse, Michael J. Lehane, Johan Esterhuizen, Inaki Tirados, Stephen J. Torr

**Affiliations:** 1 Natural Resources Institute, University of Greenwich, Chatham, United Kingdom; 2 Southern African Centre for Epidemiological Modelling and Analysis, University of Stellenbosch, Stellenbosch, South Africa; 3 The Institut de Recherche pour le Développement - The Centre de coopération internationale en recherche agronomique pour le développement (IRD-CIRAD), Bobo-Dioulasso, Burkina Faso; 4 Liverpool School of Tropical Medicine, Liverpool, United Kingdom; 5 Warwick Medical School, University of Warwick, Coventry, United Kingdom; Johns Hopkins Bloomberg School of Public Health, United States of America

## Abstract

**Background:**

Male and female tsetse flies feed exclusively on vertebrate blood. While doing so they can transmit the diseases of sleeping sickness in humans and nagana in domestic stock. Knowledge of the host-orientated behavior of tsetse is important in designing bait methods of sampling and controlling the flies, and in understanding the epidemiology of the diseases. For this we must explain several puzzling distinctions in the behavior of the different sexes and species of tsetse. For example, why is it that the species occupying savannahs, unlike those of riverine habitats, appear strongly responsive to odor, rely mainly on large hosts, are repelled by humans, and are often shy of alighting on baits?

**Methodology/Principal Findings:**

A deterministic model that simulated fly mobility and host-finding success suggested that the behavioral distinctions between riverine, savannah and forest tsetse are due largely to habitat size and shape, and the extent to which dense bushes limit occupiable space within the habitats. These factors seemed effective primarily because they affect the daily displacement of tsetse, reducing it by up to ∼70%. Sex differences in behavior are explicable by females being larger and more mobile than males.

**Conclusion/Significance:**

Habitat geometry and fly size provide a framework that can unify much of the behavior of all sexes and species of tsetse everywhere. The general expectation is that relatively immobile insects in restricted habitats tend to be less responsive to host odors and more catholic in their diet. This has profound implications for the optimization of bait technology for tsetse, mosquitoes, black flies and tabanids, and for the epidemiology of the diseases they transmit.

## Author Summary

Tsetse flies and other blood-sucking insects spread devastating diseases of humans and livestock. We must understand the host-finding behavior of these vectors to assess their epidemiological importance and to design optimal bait methods for controlling or sampling them. Unfortunately, mysteries abound in the host-finding behavior of tsetse. For example, it is strange that visual cues are more important for species found in riverine habitats, where dense vegetation restricts the range of visual stimuli, whereas olfactory cues are more important for species occurring in open savannah. To explain this paradox, we used a deterministic model which showed that restricted riverine habitats can reduce tsetse movement by up to ∼70%. This, and the fact that movement increases with fly size, can explain why savannah tsetse, especially the larger ones, rely relatively greatly on olfactory cues, are particularly available to large stationary baits, are repelled by humans, and often investigate baits only briefly without alighting on them. The results also explain why tiny, inexpensive, and odorless baits can control riverine tsetse effectively, whereas larger odor-baited devices are needed against savannah tsetse. These findings have important bearings on the study of host-finding behavior in other blood-sucking insects, including mosquitoes.

**Box 1. Method of Calculation**

An Excel spreadsheet was provided with a series of square “maps”, composed of 200×200 cells representing a total 2×2 km. If flies had to be allowed to move off the maps, each map was assumed to adjoin mirror-image maps on all four sides, so that the number of flies leaving the map at any point was equal to the number entering there. If very long bands of habitat had to be considered, the bands were fitted into the maps by making the bands take a right angle bend at intervals of nearly 2 km. Each cell had a formula which displayed a number indicating the number of flies associated with events during a step period. Starting with a map at the top of the spreadsheet, and working down through other maps below, the following stages of calculation were performed, some of which required several maps.

1. Numbers of flies present at the start of a step period.
2. Survivors of natural losses taken to occur as soon as the step period began and associated with: (i) deaths due to all causes other than starvation and (ii) feeding on hosts other than those specifically located on the maps.
3. Visual and olfactory recruitments to the immediate vicinity of specifically located baits, and the numbers surviving recruitment, before any flies stepped out of cells by the normal orthogonal dispersal. Recruitments to baits were made from the numbers of flies remaining thus far and occurred only if the step period was for host-finding, not a general step period.
4. Orthogonal dispersal of surviving flies, so that after movement the number in each cell was the number not leaving, plus the number entering from each adjacent cell.
5. As stage 3, except that it dealt with flies that had just stepped into each cell.
6. Partition of the total numbers of flies that had been recruited to the immediate vicinity of baits during stages 3 and 5, above. Flies were separated into those that: (i) responded effectively to the bait at close range and so were to be removed permanently from the population and counted cumulatively, and (ii) did not respond effectively to the bait at close range and so were to be accumulated into a temporary category considered to consist of inactive flies recovering from their recent exertions and which remained evenly distributed in good habitat within visual range of the host.
7. Number of flies available to start the next step period, and picked up at that time by the formulae of stage 1. At the end of a set of 25 host-finding steps, the numbers of flies ready to start the general steps were supplemented by flies freed from temporary inactive category mentioned under stage 6.

Calculations were controlled by the Visual Basic for Applications facilities associated with Excel and which set Excel to iterate for a number of times equal to the number of step periods required. At each iteration the calculations passed down the spreadsheet, performing stages 1–7 in succession.

## Introduction

Tsetse flies (*Glossina* spp.) occupy about ten million square kilometers of sub-Saharan Africa [1]. They feed exclusively on vertebrate blood and, in so doing, transmit those trypanosomes, namely *Trypanosoma brucei rhodesiense* and *T. b. gambiense*, that cause sleeping sickness in humans. These trypanosomes, together with others such as *T. vivax*, and *T. congolense* cause the disease of nagana in domestic animals. Host location by tsetse [2], [3] is thus a key aspect of disease dynamics. Moreover, understanding the host-orientated behavior of tsetse has led to several cost-effective means of attacking the flies [1], [4], [5], and could have implications for current and prospective methods of controlling mosquitoes, such as the use of bed-nets [6], insecticide-treated livestock [7], odor-baited traps [8] and genetically-modified vectors [9].

The various species of tsetse divide into the so called “forest”, “riverine” and “savannah” groups, of which only the latter two groups are epidemiologically important. The savannah species occupy extensive blocks of deciduous woodland and transmit mostly nagana [1]. whereas the riverine species are important vectors of both nagana and sleeping sickness and typically occur in evergreen woodland near water bodies The two groups of main vectors differ in at least four important ways: (i) savannah flies displace by an average of about 1 km/day [10], while riverine flies displace only about a third as much [11]; (ii) savannah tsetse commonly feed on large hosts such as warthog, kudu and elephant, while small animals such as lizards form much of the diet of riverine tsetse [12]; (iii) the response of savannah tsetse to odor is several times greater than for riverine tsetse [13]; (iv) savannah tsetse are strongly repelled by humans [2], whereas riverine flies are not [14], [15], [16]. These contrasts have led to marked differences between the designs of insecticide-treated screens, called targets, used to control each group [16]. For savannah tsetse the targets are 1–2 m^2^ and baited with artificial ox odor [17]; for riverine tsetse the targets are as small as 0.06 m^2^ and used without odor [18].

The distinctions between the behavior of riverine and savannah tsetse seem anomalous. For example, the avoidance of humans by savannah flies is usually attributed to the high risks of feeding on a type of host adept at killing probing insects [2], but the risks should be high for riverine flies too, so why are riverine flies not equally averse to humans? If savannah tsetse rely heavily on odor attraction, why do riverine flies not do so? Moreover, since riverine tsetse feed off small animals and land on tiny targets, why do savannah tsetse disregard such baits [19]. To explain these anomalies we hypothesized that the distinctive responses of riverine and savannah tsetse to baits is associated directly with the way that the overall size and shape of different habitats affect fly mobility, devoid of any distinctions in the innate behavior of the two groups of tsetse. This hypothesis is an extension of the experimental and theoretical evidence that various arrangements of dense bushes inside the habitat restrict the movement of tsetse and so alter the catches at baits [20], [21]. It resonates with indications from studies with other creatures that habitat geometry can be important in a variety of matters such as speciation [22], species coexistence in predator-prey relationships [23], the dynamics of such relationships [24], and population abundance [25].

While much of the behavioral impact of dense bushes within tsetse habitat has been established by experiments in the real world, involving small-scale manipulations of bush arrangements [20], [21], manipulations on a much larger and impractical scale would be required for field tests of the hypothesis that the behavior of tsetse is governed also by the overall size and shape of the habitat. Hence, we used a deterministic model to simulate within a Microsoft Excel spreadsheet the impact that the overall shape and size of habitats, together with the arrangement of bushes within them, has on tsetse displacement, catches at experimental baits, feeding success, host selection, and the efficacy of various types of target.

## Methods

### Ethics

There were no ethical issues since all work was theoretical.

### Model

The spirit of the modelling was that a cohort of flies that had started its feeding cycle moved about the habitat, encountering visual and/or odor cues from various natural or artificial baits and then fed on the baits, or was killed by them, with a probability appropriate for each bait type. Flies fed or killed at various times during the cycle were accumulated and removed from the simulation.

#### Movement

A tsetse flies for ∼25 min/day [26], at speeds of ∼24 km/h [27], giving a flight distance of ∼10 km/day. However, daily displacement is only 2–10% of this – due to the random/diffusive movement of the flies [11], [28]. This movement was modelled as a series of steps occurring within a grid of 200×200 cells, each considered to be 10×10 m, so that the whole grid represented a map of 2×2 km. At each step flies regarded as being in homogeneous terrain moved at random, from the center of one cell to the center of one of the four orthogonally adjacent cells. This model, chosen for its convenience for modelling movements between adjacent cells in an Excel spreadsheet, produces a quantitatively different rate of movement from that observed in the more traditional random walk where each step is taken in a direction chosen at random from the range 0–360°.

In the latter classical random, or diffusive, movement model, with step length *x*, the mean distance (*D*) moved from the origin after *n* steps is given by (1):
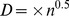
(1)When movements can be made in only four orthogonal directions, the distance moved after *n* steps is smaller. The two models can be matched, however, by setting a probability *h* (0≤*h*≤1) that a fly makes any given step. For the classical model the distance moved is now given by:
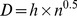
(2)For the model with orthogonal movements the distance moved (*d*) decreases as the square root of *h* so that, for a given number of steps:
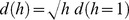
(3)The value of *h* that allows matching of the two movement models is found by equating *D* and *d*(*h*) for an arbitrarily selected step size and number of steps. Thus after 196 orthogonal steps, each of distance 10 m, the distance moved using orthogonal movements was 124.1 m when *h* = 1. Using (2) and (3) we thus require:
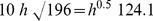
which provides a value of *h* = 0.7858 which was used in all of the modelling.

Notice that with this value of *h* the step length for the classical model, the step length is 0.7858×10 = 7.858 m. If a fly takes 1000 such steps in a day the mean distance moved will then be given by:
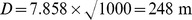
which is compatible with field estimates for riverine tsetse [11], [29].

Steps were taken as either host-searching steps, in which flies actively hunted for stationary hosts, or general steps in which flies were unresponsive to stationary baits, either because they were following a mobile bait or engaged in other activities, such as finding a resting place or larval deposition site. A set of 25 host-searching steps was alternated through the day with 25 general steps.

The inter-feed interval in tsetse averages three days [30], with a maximum of six days, during which spontaneous activity rises exponentially for five days [31]. The total number of steps was modelled as 150 on day 1 of the cycle, doubling each day to 2400 on day 5, and dropping to 1350 on day 6 when flies were close to death by starvation. The total possible number of steps per 6-day cycle was 6000.

#### Vegetation

To reflect habitat preferences, the probability of a tsetse entering a particular cell was set to 1.0, 0.1 or 0 for vegetation types defined as “good”, “poor” or “no-go”, respectively. Flies crossed between cells if the vegetation of the destination cell was as good as or better than that of the source cell. If not, the proportion crossing was equal to the probability for the destination cell divided by that for the source cell. Flies not crossing returned to the middle of the source cell. Savannah habitat was represented by large blocks of cells covered by good vegetation. Bands, or small scattered blocks, of good vegetation simulated riverine habitat. At the start of each simulation flies were distributed according to the stabilized pattern arising from vegetation arrangement alone.

#### Baits

The map was populated with two types of “bait”: (i) those located in specified cells and comprising natural hosts or insecticide-treated targets, and (ii) wild natural hosts evenly distributed over the map and which competed for tsetse with the specifically located hosts and targets. Four sizes of specifically located host were simulated; in keeping with their size they were given names of common hosts and were assigned ranges over which tsetse could detect their visual or olfactory stimuli (Table 1) based on the following rationale.

**Table 1:** Estimates of the range at which tsetse perceive hosts of various mass, using visual and olfactory cues.

Host	Mass, kg	Range, m	
		Visual	Olfactory
Lizard	2	2	6
Warthog	42	6	27
Kudu	333	11	76
Elephant	5196	28	299

The distance from which baits of roughly comparable shape can be detected visually was considered to be proportional to the bait's linear measurements. Thus, given that a model of a mammalian host, 37 cm in diameter and 50 cm long, equivalent to an animal of about 50 kg, has a visually effective range of around 6 m [32], it was possible to calculate the approximate ranges at which mammals of roughly this shape but of different body mass could be detected. For markedly elongated baits such as lizards the area covered by visual stimuli might tend to be greater than for mammals of the same body mass. Against this, lizards are often low on the ground or partly submerged in water for some of the time, and so might be visually perceptible at relatively short distance. Hence, assuming that these two opposing matters cancel each other, the formula for the range of visual perception for lizards was taken as the same as for mammals of similar mass. For all hosts larger than the lizard, the body masses chosen were such that the area of the circle in which visual perception would occur was the same as the area of a square block of a whole number of cells.

The range at which host odor can be detected depends on odor dose and the relationship between dose and plume length. The dose is likely to be related to metabolic rate, as governed by Kleiber's law [33] which states that for mammals the rate is proportional to the 3/4 power of body mass. Thus, it would be expected that the dose increases ever more slowly as mass increases. Moreover, the length of the plume is believed to increase ever more slowly as dose increases [34]. To cater approximately for both of these matters, it was taken that plume length increases as the square root of host mass. Thus, knowing that an ox of 470 kg produces a plume that attracts savannah tsetse from about 90 m [35], the plume lengths for other hosts could be estimated. Since the metabolic rate of reptiles is less than that of mammals of similar size [33], it might have been fair to adopt relatively short plume lengths for lizards. However, this was not done, so perhaps over-estimating the true range of perception of lizard odor. Consequently, the model's indication that lizard odor is poorly effective is likely to be safe.

**Figure 1:**
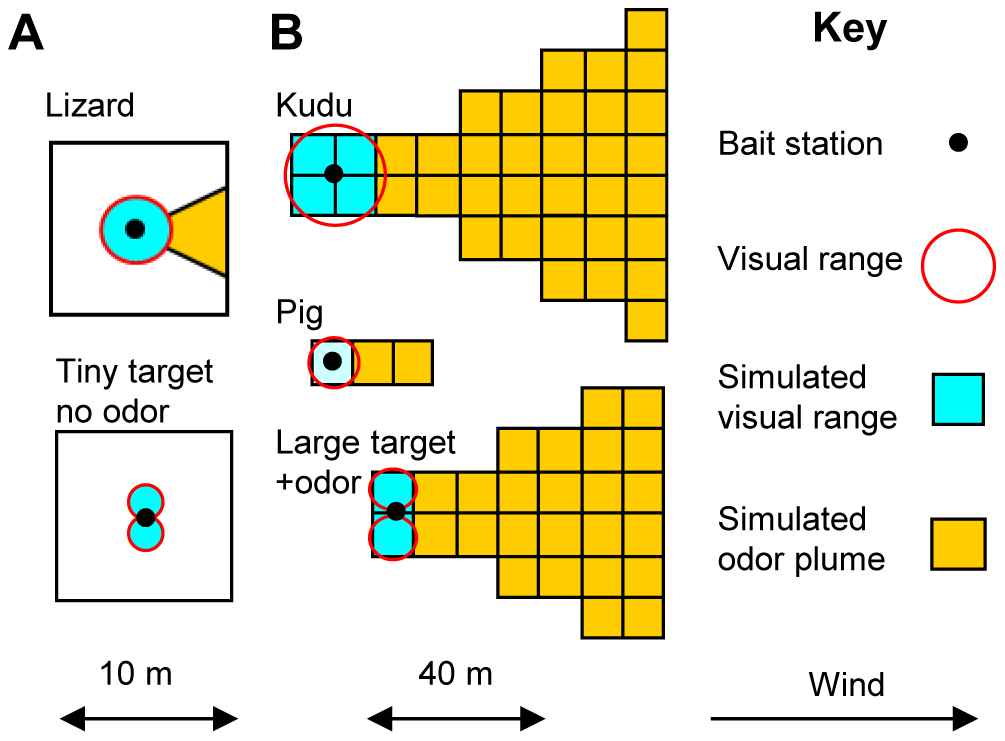
Simulated areas covered of visual and olfactory stimuli. A: areas within a single cell around a lizard and tiny odorless target. B: groups of cells around and near a kudu, pig and large target used with odor.

The cells receiving host stimuli were simulated as shown in Figure 1, involving odor plumes considered to extend downwind as a triangle, with the edge of the plume deviating 26° from the axis. Cells considered to contain the plume were fitted as closely as possible to the triangle. Targets were large or tiny, and assumed to have the ranges of visual effectiveness of a kudu and lizard, respectively. The area of visual perception was adjusted to allow for the two dimensional form of targets; the range of olfactory detection for large targets used with odor was 60 m (Fig. 1).

#### Stimulation, recruitment and death

All flies in each cell receiving visual and/or olfactory stimuli from either the pig, kudu or elephant were considered to be stimulated since such cells were taken to be covered completely by stimuli. With the lizard, whose stimuli were regarded as covering less than a whole cell, it was taken, arbitrarily, that 50% of the flies in the cell were stimulated when only visual stimuli were offered, and 80% when odor was also provided. For the tiny target, which could be perceived visually from only two directions and was always used without odor, it was taken that 25% of the flies in the cell were stimulated.

In each host-searching step period, all flies initially present in the area where stimuli from specifically located baits were perceptible, and flies moving into that area, transferred immediately to the vicinity of the bait itself. Of the flies that arrived in that vicinity, a certain fraction (***f***) showed an effective response to it, *i.e.*, either feeding on it or being caught or killed at it, before the end of the period. Such flies were removed permanently from the population and their numbers were accumulated. For simulations of catches at natural hosts, it was considered that the hosts were placed singly in a pen of netting that electrocuted arriving flies [2]. In these cases the value of ***f*** was 0.6, according with estimates of the capture efficiency of the netting [36]. For studies of feeding, ***f*** was 0.1 on day 1, rising by 0.1 each day to be 0.6 on day 6, in keeping with evidence that the probing responsiveness rises linearly during the hunger cycle [37]. These values of ***f*** were adopted also for studies of target performance, so allowing that: (i) not all of the flies visiting a bait actually contact it, especially when the flies are in the early part of their hunger cycle [2], [38], and (ii) the insecticide deposit on the targets is unlikely to be perfectly efficient all of the time. Flies not showing an effective response (1-***f***) were accumulated into a separate temporary category in which they were considered to be unresponsive to the bait while they recovered from their recent exertions at it. These flies re-joined the main population after the last host-searching step of each group of 25 such steps. They were then released evenly into those cells of good habitat in which visual stimuli occurred, so that general steps caused them to diffuse away from the bait station – the flies being unresponsive to the bait until the next group of host-searching steps.

When the specifically located baits were objects introduced artificially for experimental or control purposes, they competed with wild natural hosts. Tsetse visiting such wild hosts had the same probing responsiveness as above. Thus, given an input for the probability of finding a wild host in any step period, it was possible to calculate the removal of flies by these hosts. The input was set at 0.00125, the value identified by Excel's Goal Seek as producing a hunger cycle lasting the required average of three days in the absence of any introduced bait. In such circumstances, the mean death rate by starvation was 2.7% per cycle, modelled as occurring at the end of the sixth day. Consistent with field indications [39], the mortality of tsetse due to all causes other than starvation was modelled at 3% per day, distributed as a survival rate over each step.

## Results

### Movement in blocks and bands of habitat

**Figure 2:**
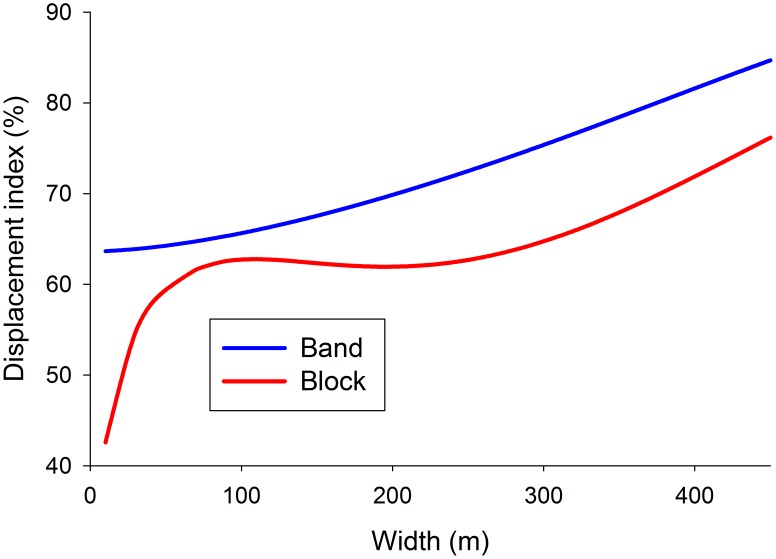
Effect of band and block width on movement. Mean displacement after 1000 steps in landscapes in which good habitat was restricted to various widths of bands surrounded by no-go area, or to square blocks in a checker-board with poor habitat. Displacement is expressed as a percent of the displacement in a large block of homogeneous, good habitat.

The ability to find stationary baits depends largely on displacement rate [19]. The principles applying to this rate were elucidated by seeding flies in the central cell of a band or block of good habitat and allowing them to execute the average daily allocation of 1000 steps, in the absence of natural death or removal by baits. Blocks were in a checker-board arrangement with poor habitat so that flies could diffuse between blocks of good habitat, albeit slowly. Bands were flanked by no-go areas to focus only on movement within the band. The results with different widths of blocks and bands indicate that at widths of 10 m the displacement was only 43–64% of the displacement in homogeneous habitat (Fig. 2). The figures increased with increasing widths, but were still only 76–85% at widths of 450 m. At any given width, the displacement in a block was less than in a band. The complex curve for blocks was associated with the change in the ratio of perimeter to area, and hence the proportion of flies located where they could step out of the block.

### Heterogeneity within habitats

**Figure 3:**
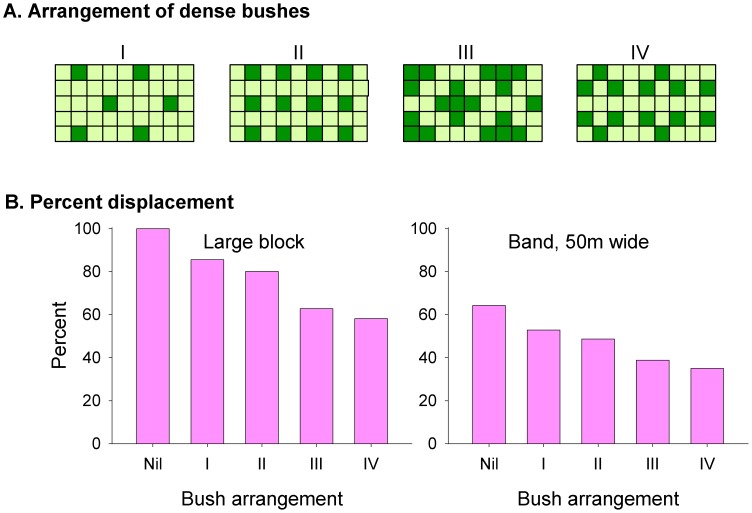
Effect of bushes on movement. A: various arrangements of bushes in sections of a band of habitat 50 m (5 cells) wide, surrounded by no-go area. B: displacement after 1000 steps with no bushes (Nil) or bushes in arrangements I–IV, in good habitat consisting of a large block or a band 50 m wide. Displacement is expressed as a percent of the displacement in a large block of good habitat containing no bushes.

To assess the effect of heterogeneity within the overall shapes of habitats, cells of no-go vegetation simulating impenetrable bushes [20], [21], were located within habitats of various shape. Findings from simulations with a variety of bush arrangements are exemplified (Fig. 3) by data for a 50 m-wide band with either no bushes, or each of four different bush arrangements, and for a large block composed of such bands placed parallel and adjacent to each other, with the adjoining parts of each band being mirror images. The rate of displacement tended to decline as: (i) numbers of bushes increased, (ii) flight paths between dense vegetation became more tortuous, and (iii) the abundance of dead-ends rose, so that the flies expended much flight on retracing their steps. Although real bushes in the field are unlikely to show the sort of serially repeated arrangements modelled above, the overall effects are likely to be similar.

Allowing that riverine habitat occurs in bands or small blocks, and is often more densely bushed than savannah, the above results match field observations that tsetse displacement is greatest with savannah tsetse [10], [11], [39]. For simplicity, subsequent modelling assumed that all habitats contained no dense bushes. With that assumption the differences found between the efficacy of baits in riverine habitats and large blocks of savannah tend to be conservative indications of real differences.

### Simulated field experiments

The relative importance of visual and olfactory stimuli is commonly estimated in the field by comparing catches from a host animal with those from an odorless model animal of the same size [2], [40]. In simulating such experiments, the two types of bait were operated for six days in a crossover design, alternating between sites that were sufficiently far apart to ensure that the baits there did not compete with each other. The baits were present for half of the daily step periods each day, consistent with the fact that field catches of tsetse are often made in the afternoon only [2]. The simulated catch with each bait was expressed as a percent of the initial abundance of tsetse per square kilometer of the good habitat, and the efficacy of odor relative to visual stimuli was taken as the percent by which the addition of odor increased the catch above that with visual stimuli alone. As expected, catches and odor efficacy increased with bait mass (Table 2). Intriguingly, catches declined markedly on going from the large block of habitat to the bands, but the decline was greatest with the large baits and when odor was used. Consequently, bait size was relatively unimportant in the bands, and the percent efficacy of odor in the narrowest band was around a quarter of the efficacy in the large block. Similar indications were produced when the baits were operated in habitat restricted to small blocks. For example, when the block consisted of just one cell, the catch with the lizard was >99.9% of the catch with the elephant and percent efficacy of odor was <0.1% with either animal.

**Table 2:** Simulated catches of tsetse from an electric pen with hosts of various mass in different habitats.

Habitat	Lizard	Pig	Kudu	Elephant
***Catch with visual stimuli alone***				
Large block	0.231	0.265	0.434	0.950
Band, 50 m	0.154	0.158	0.188	0.240
Band 10 m	0.047	0.046	0.044	0.048
***Catch with visual stimuli + odor***				
Large block	0.264	0.461	1.400	5.592
Band, 50 m	0.174	0.254	0.409	0.756
Band 10 m	0.049	0.060	0.082	0.151
***Relative efficacy of odor (%)***				
Large block	14.5	73.9	222.4	488.7
Band, 50 m	12.8	61.2	117.5	214.6
Band 10 m	4.3	30.6	85.9	214.6

Catches are expressed as a percent of the initial population per square kilometer. Relative efficacy of odor is the percent by which the catch with visual stimuli plus odor exceeds the catch with visual stimuli alone.

Outputs for the percent efficacy of odor in the large block accord well with field data for savannah flies. For example, for *G. m. morsitans* and *G. pallidipes* in the field, the relative efficacies of odor with an ox (454 kg), donkey (204 kg) kudu (136 kg) warthog (82 kg) and bushpig (73 kg) averaged 435%, 175%, 89%, 56% and 73%, respectively [2]. More remarkably, outputs for the bands or small blocks accord well with the limited field efficacy of odor against riverine tsetse [13], despite the model's provisions that the innate responsiveness and mobility of flies in the bands was exactly the same as in the large block. Hence, habitat geometry, irrespective of any innate behavioral distinctions, can account for most differences between patterns of field catches of savannah and riverine tsetse.

### Efficacy of targets

**Figure 4:**
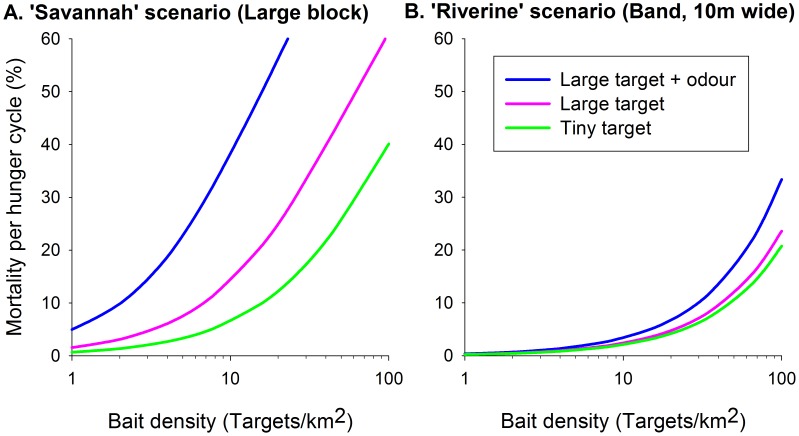
Efficacy of various targets at various density. Percent of the tsetse population killed per hunger cycle by three different types of target at various densities, in a large block of habitat (A) or in a band 10 m wide (B).

Simulations were made with various densities of large and tiny targets (Fig. 1) operated continuously in a large block or 10 m-wide band. As in field campaigns against riverine tsetse, tiny targets were used without odor, but large targets were modelled with and without artificial ox odor, according with the field use of large targets against savannah and riverine flies, respectively. In keeping with field catches at targets [17]–[19], the numbers of targets required to achieve a given rate of kill differed greatly between the large block and the band (Fig. 4). To interpret the outputs it can be taken that an imposed death rate of about 4% per day, or 12% per feeding cycle, reduces field populations of tsetse by 99.99% per year, leading to population elimination in the absence of invasion [16]. On that basis, outputs accord with field indications for the numbers of various sizes of target needed to control savannah [41] and riverine [42] tsetse, and for the efficacy of odor with targets in savannah [2] and riverine [43] habitats. Hence, the results offer further support for the hypothesis that habitat geometry, not differences in innate behavior, determines much of the distinctive availabilities of riverine and savannah tsetse.

### Feeding success

To explore the abilities of various sizes and population densities of hosts to support the tsetse population, it was assumed that flies fed only on those stationary hosts that the model introduced, so no allowance was made for feeding on any other animals. Feeding success was scored after four days when fed flies had replenished their food reserves after an average of around three days, *i.e.*, the normal length of the hunger cycle. It was also scored after six days, when flies were about to die of starvation. Since some flies died of causes other than starvation, percent feeding success could not reach a full 100%.

**Figure 5:**
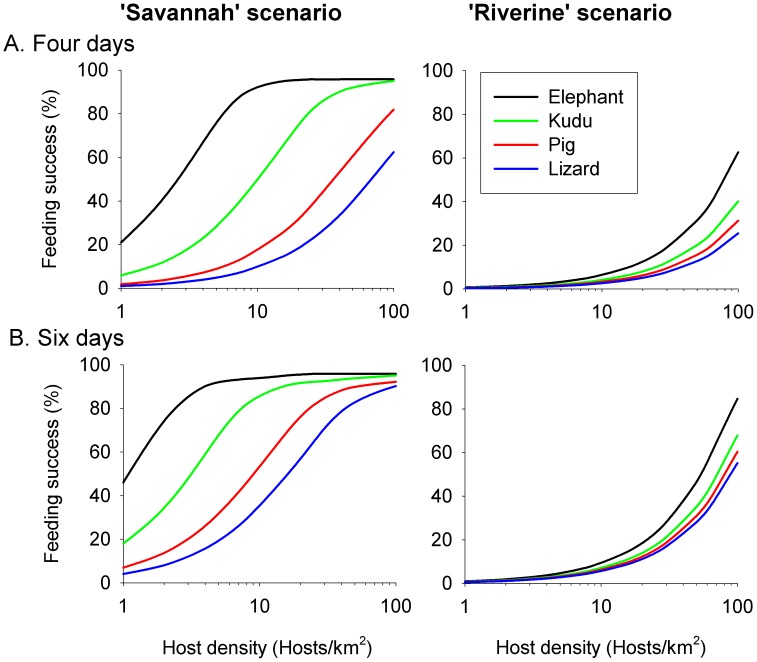
Feeding success with various hosts at various density. Cumulative percent of tsetse that had fed after four days (A) or six days (B), in a large block of habitat or in a band 10 m wide.

As expected from the above work with targets and simulated field catches, the host numbers required to allow a given level of feeding success were much greater in a narrow band than in the large block, and the efficacies of the various hosts differed greatly in the block but relatively little in the band (Fig. 5). Thus, in the large block, about 15–30 lizards led to the same feeding success as one elephant, but in the band only about 2–3 lizards were required.

As in other modelling [44], the number of flies discovering hosts decreased substantially when hosts were grouped instead of being singly and evenly distributed. Consider, for example, a population of lizards at an overall density of 100/km^2^ in a band of habitat 10 m wide. When the lizards were distributed singly and evenly the 4-day feeding success was 25%, but dropped to only 2% when the lizards occurred in evenly distributed groups of four, with each group involving a lizard in each cell of a line of four cells along the axis of the band of habitat. In a large block of habitat the comparable figures for feeding success were 65% for lizards distributed singly, as against only 11% for the grouped lizards.

The outputs (Fig. 5) are consistent with the abilities of known host populations to support tsetse. Thus, savannah tsetse at Sengwa, Zimbabwe, were maintained by a mixed population of hosts comprising an average of ten warhogs, plus two elephants and several kudu and other bovids per square kilometre [45]. Moreover, the model's indications that tsetse in restricted habitats can be supported largely by small hosts such as lizards, with population densities of around 50–100/km^2^ [46], agree with the frequency of lizards and other small creatures in the blood-meal identifications of riverine tsetse [12].

### Fly mobility and host selection

Mobility has thus far been assumed to be the same for all flies. However, female tsetse displace at a greater rate than males [10]; young flies with poorly developed flight muscles [47] and old flies with damaged wings displace relatively little, and daily flight times can double or halve according to seasonal temperature [26]. To simulate this variability, the daily number of flight steps was increased or decreased threefold.

As expected, the greater the mobility of flies the sooner they fed. However, it was more instructive to consider what this implied about the extent to which flies could afford to be selective about feeding on hosts they encountered. To explore this, the model's map was provided with an even spread of hosts. At different points in the feeding cycle, calculations were then made of the probability that flies that did not feed at that point would die of starvation. In any given habitat, and with any given size and abundance of host, this probability increased with the number of host-searching days completed. It increased also with a reduction in the number of step periods allowed per day and was greater in the narrow band than in the large block. The latter phenomena are illustrated by considering outputs with kudu at 16/km^2^, which represents roughly the abundance and mean size of the main hosts, *i.e.*, warthogs, elephants and kudu, that sustained the tsetse population in the savannah at Sengwa [45], discussed above. Simulations were also made with host populations consisting of lizards at 100/km^2^, to be closer to a host situation more typical of riverine habitats [46].

The results show that tsetse in large blocks of habitat can afford to feed much more selectively than when they are in a restricted habitat carrying the same types and abundance of hosts (Table 3). The comparison between real riverine and savannah areas will depend crucially on the numbers and sizes of hosts present in each situation, and on the intrinsic mobility of the tsetse present. However, the principles are established that a reduction in the innate mobility of tsetse, and the limits that restricted habitats impose on host location, can greatly favor a strategy of feeding on any host encountered.

**Table 3:** Percent probability that flies will die of starvation under various conditions.

Hosts	Habitat	Days completed	Steps per cycle		
			2000	6000	18000
Kudu 16/km^2^	Large block	2	16.6	0.5	0.0
		5	54.9	16.6	0.5
	Band 10 m wide	2	93.4	89.1	81.9
		5	98.9	98.1	96.6
Lizards 100/km^2^	Large block	2	16.9	0.5	0.0
		5	52.6	14.5	0.3
	Band 10 m wide	2	68.9	40.1	8.0
		5	91.0	78.8	50.2

Flies are exposed to different host populations, in different habitats, on different days of the hunger cycle, and are able to execute various numbers of steps per cycle.

## Discussion

The host-oriented behavior of tsetse is arguably better understood than that of any other blood-sucking insect [13], [48], allowing models of bait-finding to employ a wealth of empirical data as inputs and for output validation. Our model indicates that distinctions between riverine and savannah tsetse in respect of daily displacement and availability to various sizes of visual bait and odor plume are due largely to the immediate circumstantial effects of habitat geometry, rather than evolved differences in innate behavior. This indication must arise with any model that approaches reality since output patterns will be set by the following five principles. First, in restricted habitats the full benefit of stimuli from large baits is lost because some of the ambit of the stimuli covers places devoid of flies. This problem is especially severe with small blocks, as against bands, since the stimuli can go out of the block on all four sides. Second, even if stimuli from large baits do not go out of a small patch of habitat, the effective advantage of seeking large hosts is reduced because random flight within the patch ensures that a relatively small host there can be discovered before long. Third, the more restricted the space that tsetse occupy the less readily can they diffuse from their start point, so reducing their probability of finding a distant bait. Fourth, at any given density of baits, the more attenuated the habitat the greater the mean distance between flies and the nearest bait. Thus, if bait density is 100/km^2^ the average distance between flies and the nearest bait in large blocks is about 40 m, as against 250 m in a band 10 m wide. Likewise, an extensive ambit of bait stimuli can reduce substantially the mean distance the flies must displace to detect the bait in the large block, whereas it can reduce this distance in the band by relatively little. Finally, the time taken to travel any given distance by diffusive movement is proportional to the square of the distance [11].

Despite the immediate importance of habitat geometry, different species are likely to have evolved some innate behavior patterns suiting the distinctive demands of finding food in their particular environments. Any innate differences might relate not so much to means of locating hosts but rather to the response adopted after discovering hosts of various type, particularly men as against more tolerant, and less dangerous, hosts. Modelling suggests that the high mobility of tsetse in homogeneous and extensive habitats, and the comparative ease of finding hosts there, means that unless savannah tsetse are about to die of starvation they should be anthropophobic, in accord with field observations [2], [3], [49]. The corollary is that the anthropophily of riverine tsetse [15] is due to the poor mobility of flies in restricted habitats and the associated difficulties of finding safer hosts. In any event, the less a fly displaces the more important it is to investigate any host thoroughly before rejecting it, implying that in such circumstances the flies will remain longer with a host and be less discerning about alighting on it. Moreover, flies with low movement rates must rely on ‘ambushing’ passing hosts, as against active searching.

### Unification

Our results suggest the possibility of reducing the wide variety of host-orientated behavior to a unifying framework applicable to both sexes and all species of tsetse in all habitats, including the many forest-group species not modelled here. The development of such a framework requires further theoretical and experimental attention. Nevertheless, host location must depend largely on displacement rates which affect: (i) effectiveness of odor attraction, (ii) reliance on small, abundant and solitary hosts, (iii) performance of small targets relative to large, (iv) repellence of humans, (v) importance of stationary as against mobile baits, and, (vi) persistence near hosts and the strength of alighting responses.

The magnitude of each of these phenomena is expected to be governed by (i) the width and length of the overall habitat, (ii) proportion of habitat that allows free flight, (iii) fly size, since innate displacement potential increases with size and (iv) proportion of the fly's energy available for flight [47]. Host-finding is likely to be influenced also by parameters other than those governing displacement. For example, changes in vegetation affect the length and structure of odor plumes [50], [51]. Nonetheless, the above four parameters, among which habitat geometry seems very important, could go far towards rationalizing much of the apparent variety of tsetse behavior. Empirical support for a unifying framework is provided by results from three sources.

First, some of the most comprehensive data for savannah tsetse come from Rekomitjie, Zimbabwe. The biggest fly present, female *G. pallidipes*, is twice the size of the smallest, male *G. m. morsitans*. In accord with expectation, the larger flies are the most mobile [10], the most available to stationary odor baits, the most repelled by humans [2], the least available to tiny, as against large, targets [19], the least persistent and the least likely to alight [2].

A second source of support is provided by several studies of tsetse that occupy habitats atypical of their group. Thus *G. longipennis*, of the forest group, occupies savannah and in keeping with its large size and habitat, is as mobile as *G. pallidipes* [52], is repelled by humans and readily available to host odor [53]. In expected contrast, G. *brevipalpis*, a large forest species which has remained in forest, is less available to odor [54]. The smallest tsetse, *G. austeni*, is a savannah-group fly found in coastal thickets. In accord with its small size and dense habitat, its availability to odor is much less than for other savannah species [54]. The riverine fly, *G. tachinoides*, lives in relatively open habitats and is relatively responsive to odor [55], albeit not as much as other tsetse living in savannah – as predicted since it is smaller than such tsetse.

Third, and perhaps the most telling, studies of the riverine tsetse, *G. fuscipes fuscipes*, near Lake Victoria in Kenya, showed that adding odor to traps was ineffective in narrow (5–10 m wide) forest habitats but doubled catches in a larger block of forest covering 1.4 km^2^ [56]. Presumably, the closeness of the habitats ensured that they contained flies with the same innate responsiveness.

### Further research

While the outputs of the model and the predictions of the unifying framework fit well with existing field data, there is a need for new field experiments specifically aimed at confirming and extending present indications. For example, it would be particularly informative to elucidate the response of riverine species of tsetse to visual and olfactory stimuli under circumstances not expected to limit the expression of such responsiveness. One approach would be to study further the behavior of riverine tsetse in large blocks of woodland [56]. Another approach is suggested by the expectation that the catches in the first few minutes of the exposure of a bait depend primarily on the responsiveness of flies already in the ambit of the bait's stimuli, whereas the later catches are governed by the way that habitat size and shape govern the rate at which tsetse diffuse into that ambit from far away. Hence, to highlight the basic responsiveness to bait stimuli in habitats that reduce fly diffusion, it would be pertinent to accumulate the catches of a bait that appears for brief periods interspersed with longer periods in which the baits are hidden while flies move in to re-populate the vicinity [20]. The time needed to produce such re-population would itself be of interest in indicating the rates of fly movement [10]. A further approach would be to use a bait that moves to a succession of stations a short distance apart, stopping at each just long enough to recruit flies from the area covered by the odor plume. Indeed, such minor movement and stopping would come closer than any research yet done to duplicate the common behavior of natural hosts.

### Practical implications

The simulations offer support for using tiny odorless targets to control riverine tsetse in restricted habitats [18] but warn that in broader habitats such as those that can occur in mangrove ecosystems, a larger target with odor might be more cost-effective. Our results confirm that relatively high densities of targets are needed per unit area of habitat to control riverine tsetse, but these high densities are offset by the fact that such habitats cover a small proportion of the land surface. Thus, in places where people and livestock need to be protected against disease during visits to infested localities, the target density required per total land surface tends to be small, at around 7/km^2^ (Torr and Lehane, unpublished).

While aversion to humans seems to be the main reason why savannah tsetse are minor vectors of sleeping sickness today, they might become more important if climatic or anthropogenic change restricts tsetse habitat.

The relationship between habitat and host-finding in tsetse is likely to apply to other blood-sucking insects. While data are less extensive for other insects, there are indications that differences are consistent with expectations. For instance, horse flies, stable flies, and blackfly living in extensive woodlands [48] are highly responsive to host odors whereas in riverine habitats near Lake Victoria these species show the same type of pattern as for tsetse in riverine [56]. Malaria mosquitoes inhabiting savannah woodland (*Anopheles arabiensis*, [40] and extensive wetlands (*Anopheles melas*, [57], [58]) are also highly responsive. On the other hand, bird-biting species of *Culex* [59], and *Aedes aegypti* (the vector of dengue virus) in urban settings [60], seem much less responsive. We suggest that the restricted and heterogeneous habitats of tree canopies and urban environments reduces mobility in much the same way that riverine habitats affect tsetse. Field studies to explore this hypothesis could provide important new insights into the transmission dynamics and control of West Nile and dengue viruses transmitted by *Culex pipiens* and *Aedes aegypti*, respectively.
